# In vitro evaluation of different implant systems and their influence on primary stability

**DOI:** 10.1038/s41598-026-35112-5

**Published:** 2026-01-09

**Authors:** Osmar de Agostinho Neto, João Victor Frazão Câmara, Anton Schestakow, Amanda de Oliveira Pinto Ribeiro, Tamara Teodoro Araujo, Bruno Salles Sotto-Maior

**Affiliations:** 1 Faculty of Dentistry, Federal University of Rio de Janeiro, Rio de Janeiro, Brazil; 2 Saarland University, Homburg, Saar, Germany; 3 Bauru School of Dentistry, University of São Paulo, Bauru, SP Brazil; 4 Faculty of Dentistry, Federal University of Juiz de Fora, Juiz de Fora, Brazil

**Keywords:** Dental implant, Torque controller, Milling, Oral implantology, Engineering, Health care, Medical research

## Abstract

The aim of this in-vitro study was to compare the primary stability and bone quality changes of three milling systems for implants. Osteotomies were performed on fresh, low-density bovine ribs using three different milling techniques for the Strong SW implant system: conventional drilling according to the manufacturer’s protocol (control), osseodensification, and bone expander (n = 5 per group). Prior to implant insertion, bone quality at the cervical, body, and apical regions was assessed using micro-computed tomography. Implants were then placed at the bone level, and primary stability was evaluated by measuring insertion torque with a digital torque meter. Bone quality and insertion torque values were statistically analyzed using one-way ANOVA followed by Tukey’s multiple comparison test (*p* = 0.05). There were no statistically significant differences between the cervical, body, and apical regions, as well as among the implant groups SIN, VERSAH, and MAXIMUS (*p* < 0.05). Furthermore, the insertion torque of the SIN group (35 ± 21.5 N/cm), VERSAH group (43.2 ± 27.1 N/cm), and MAXIMUS group (59.6 ± 28.5 N/cm) also showed no statistically significant differences (*p* < 0.05). These findings suggest a similarity in bone microarchitecture and insertion strength among the different implants studied. The three milling techniques demonstrated comparable performance, showing no significant differences in bone microarchitecture or primary stability. These results indicate that all systems provided similar conditions for implant insertion in low-density bone.

**Supplementary Information:**

The online version contains supplementary material available at 10.1038/s41598-026-35112-5.

## Introduction

Contemporary implantology has seen significant advancements in both the quality of materials and surgical techniques, aiming to achieve more predictable outcomes during the surgical phase^1^. In recent years, there has been substantial evolution in the macro and micro-geometric design of implants, with the goal of improving mechanical interlocking in various clinical conditions^2^. These innovations are essential for optimizing primary stability, a critical factor for the long-term success of the implant.

Primary stability refers to the mechanical anchorage of the implant within the bone, and its achievement depends on several factors, including bone volume and quality, the implant design, and the surgical technique used^3^. Primary stability is commonly assessed through insertion torque during implant placement. When this torque exceeds 30 N/cm, the implant is considered suitable for immediate loading, which is essential for immediate loading protocols, where the initial success can significantly impact the osseointegration process^4^. This clinical parameter is widely used to determine the timing of prosthetic activation, especially in immediate loading cases^5^.

The evolution of bone preparation techniques has aimed to optimize primary stability, with undersized milling (subinstrumentation) being one of the most commonly employed approaches, which aims to preserve bone while enhancing mechanical anchorage^6^. However, a promising alternative that has gained attention is bone expansion milling, which aims to preserve bone while increasing its density at the surgical site^7^. The osseodensification technique, performed using Densah Burs (Versah LLC, Michigan/USA), compacts the bone without removing tissue, increasing the bone-implant contact area (BIC) and implant stability quotient (ISQ) values, contributing to greater primary stability and reduced healing time^8,9^. These advancements have demonstrated benefits in increasing primary stability, facilitating the osseointegration process, and leading to faster and more effective recovery^10^.

However, challenges still remain in achieving stable osseointegration in low-density bone areas, such as those commonly found in the maxilla or trabecular bone. In regions of low-density bone, achieving adequate primary stability is particularly challenging. Micromotion of the implant becomes a considerable risk, as the lack of robust mechanical locking can disrupt the formation of a stable osteogenic environment, delaying or even preventing osseointegration^11^. Furthermore, these regions often suffer from reduced vascularization, which impairs the supply of essential nutrients and growth factors needed for bone healing. Reduced vascularization prevents the proper recruitment of osteoblasts and the formation of a stable bone-implant interface, delaying osseointegration^12,13^.

Another critical factor in the early phases of osseointegration is the inflammatory response^13^. The bone’s immune response in low-density regions may be less efficient, increasing the risk of prolonged inflammation and infection. Chronic inflammation can hinder osteogenesis, interfering with the healing process and increasing the risk of implant failure^13^. Additionally, excessive mechanical loading on implants, especially in low-density bone areas, can be detrimental. The lack of sufficient bone density to resist external forces may result in implant micromotion during the healing phase, interfering with the formation of the bone-implant interface and thus hindering osseointegration^11,14^. Beyond the anatomical characteristics of the bone, patient-specific factors, such as osteoporosis, diabetes, or other metabolic bone disorders, may further complicate osseointegration, particularly in low-density bone areas. These conditions can negatively affect bone quality, slowing or preventing bone remodeling and, consequently, implant integration.

In addition to optimizing surgical techniques and implant design, implant surface modifications play a pivotal role in enhancing osseointegration, particularly in low-density bone areas. One of the most effective approaches to improve the bioactivity of implant surfaces is the incorporation of calcium phosphates (CaPs), such as hydroxyapatite (HA) and tricalcium phosphate (TCP), which have a mineral composition similar to that of natural bone. These biocompatible materials promote osteoblast adhesion, proliferation, and differentiation, facilitating the formation of a mineralized bone layer around the implant and increasing the BIC^15,16^. Surface modification with calcium phosphates has been shown to accelerate the osseointegration process, particularly in low-density bone, by enhancing primary stability and reducing healing time.

Therefore, the aim of this study was to compare, in vitro, three implant systems: (a) burs of the implant system (control), (b) osseodensification burs (Densah Burs) and (c) Bone Expander burs (Maximus) in order to analyze their influence on primary stability. The null hypothesis tested was that there would be no significant difference in primary stability between the implant systems used.

## Material and methods

### Materials and experimental groups

This experiment was carried out in two laboratory methodologies using fresh bovine rib specimens as they have low density characteristics, an important point for the instruments evaluated in this study. To prepare the implant osteotomy in the control group, standard drilling methods were followed. The sequence began with a 2.0 mm spear drill, followed by a 2.0 mm twist drill and ended with a 3.0 mm conical design drill, all instruments being from the SIN STRONG SW system (SIN Strong CM, São Paulo). This implant system has CM 11.5 and 16°. The irrigation was used for all drilling procedures. In the milling process of the SIN implant system, the protocol follows the system’s milling sequence, with 1200 rpm for the initial drill and 800 rpm for the subsequent drills. In the Maximus system, milling is done at 800 rpm in the clockwise direction. In the Versah system, milling is performed at 800 rpm, with the initial drill rotating clockwise (cutting) and the subsequent drills rotating counterclockwise (expansion).

**Fig. 1:**
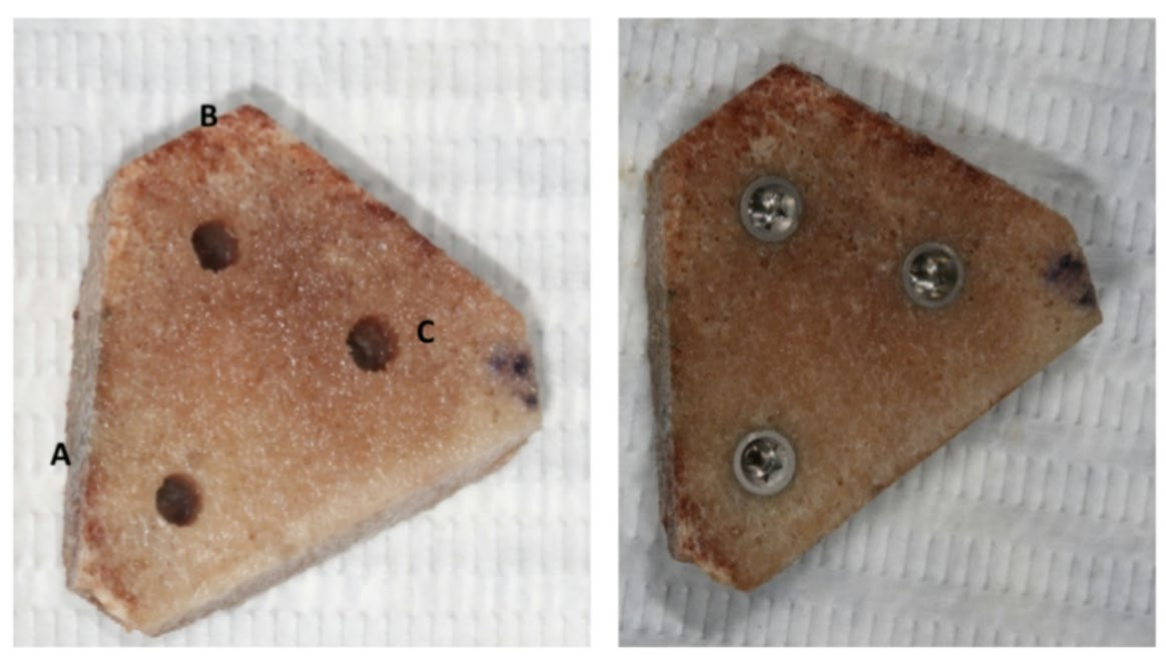
Bovine ribs after milling with each instrument (A—SIN drills, B—Densah drills, and C—Bone Expander reamers), followed by the installation of the implants.

For the experimental group using the osseodensification system (Densah Burs—Versah LLC.—Michigan/USA), milling was started with a 1.7 mm pilot drill in a clockwise direction (cutting mode) and followed by a series of new multiple conical drills in a counterclockwise direction (osseodensification mode) with diameters of 2.0 mm (VT1525) and 3.0 mm (VT2535). In the experimental group, the Bone Expander system (Maximus Produtos Odontológicos—Minas Gerais/Brazil) was used in a clockwise direction, starting with a 1.3 mm pilot cutter and followed by bone reamers in progressive order up to the indicated size (ALO34.TI) for installing the implant. The bovine ribs after milling with each instrument are shown in Fig. 1.

### Bone microarchitecture evaluation

**Fig. 2:**
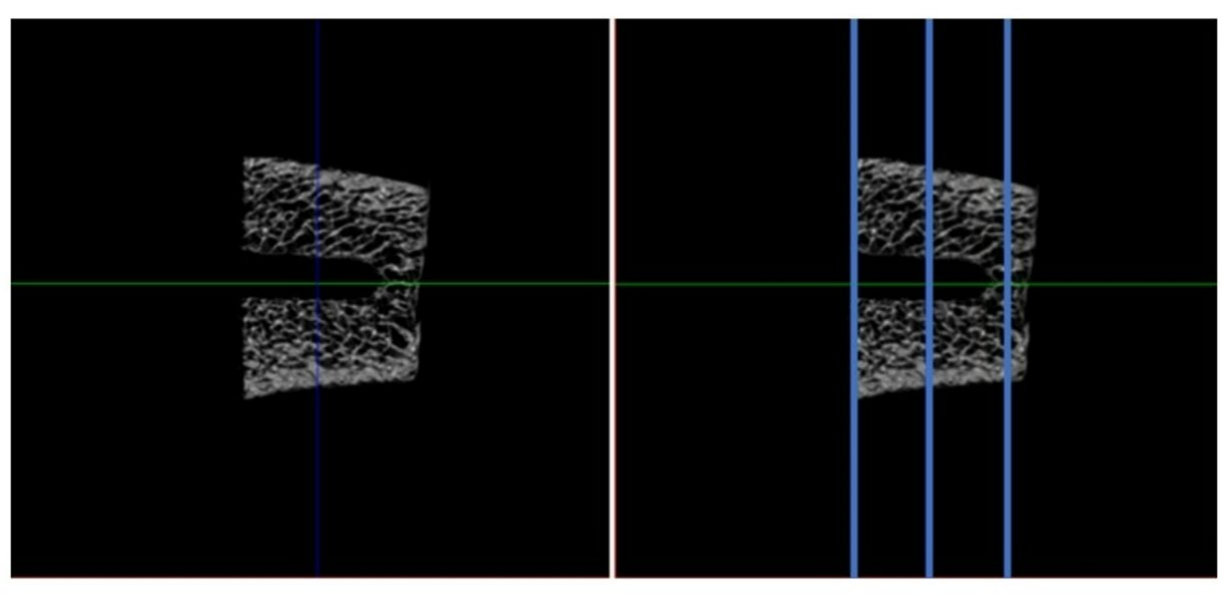
Micro-CT evaluation. Representative axial microtomographic slice from one of the drilling protocols, showing the delineation of the analyzed regions: cervical, body, and apical.

A total of five milling operations (n = 5) were carried out for each of the following systems: (1) Implant system milling cutter (SIN Strong CM, São Paulo/Brazil—control group), (2) Osseodesification milling cutter (Densah Burs—Versah LLC.—Michigan/USA) and (3) Bone Expander bone reaming cutters (Maximus Produtos Odontológicos—Minas Gerais, Brazil)). All the milling operations followed the parameters provided by the manufacturers for working with a Strong SW CM 3.5 × 8.5 mm implant (SIN Strong CM, São Paulo) in a low-density bone and always at bone level. A microtomography image was then taken on the Skyscan 1172 (Skyscan, Antwerp, Belgium) for each bone sample with a nominal isotropic voxel size of 8 μm (X-ray source 92 kV, 120 mA) (Fig. 2). A 180° rotation step and a 0.5 mm aluminum filter were used to reduce noise. The bone microarchitecture was analyzed at three points in the axial plane for each milling: axial, middle and apical. A mask of 500 μm from the center of each milling was applied to limit the area analyzed. Bone surface area (pixel^2^) and volume (pixel^3^) were analyzed, and the surface-to-volume ratio was calculated and statistically evaluated.

### Assessment of insertion torque

Considering that the surgical instruments being tested are indicated for the creation of an implant socket by a non-subtractive technique, but rather by bone expansion, in addition to the evaluation in the microtomography image exams, it was suggested that osseointegrated implants be installed to check initial stability by measuring the peak insertion torque using an electronic torque wrench. Thus, the fresh bovine ribs were milled eight times (n = 8) for each technique described above. After milling, each socket received a Strong SW CM 3.5 × 8.5 mm osseointegrated implant (SIN Strong CM, São Paulo) at bone level. Subsequently, using an INSTRUTHERM TQ-680 digital torque wrench (São Paulo, Brazil), each implant had its initial locking measured. The values obtained were tabulated and submitted to statistical analysis.

### Statistical analysis

The bone surface/volume ratio calculated in the microtomography according to the milling technique and region (cervical, body or apex), and the peak insertion torque values passed normality with Shapiro–Wilk test, and differences were analyzed using analysis of variance (ANOVA) and the Tukey test (*p* < 0.05) with GraphPad Prism 10 (GraphPad Software, Boston, USA).

## Results

### Bone microarchitecture

**Fig. 3:**
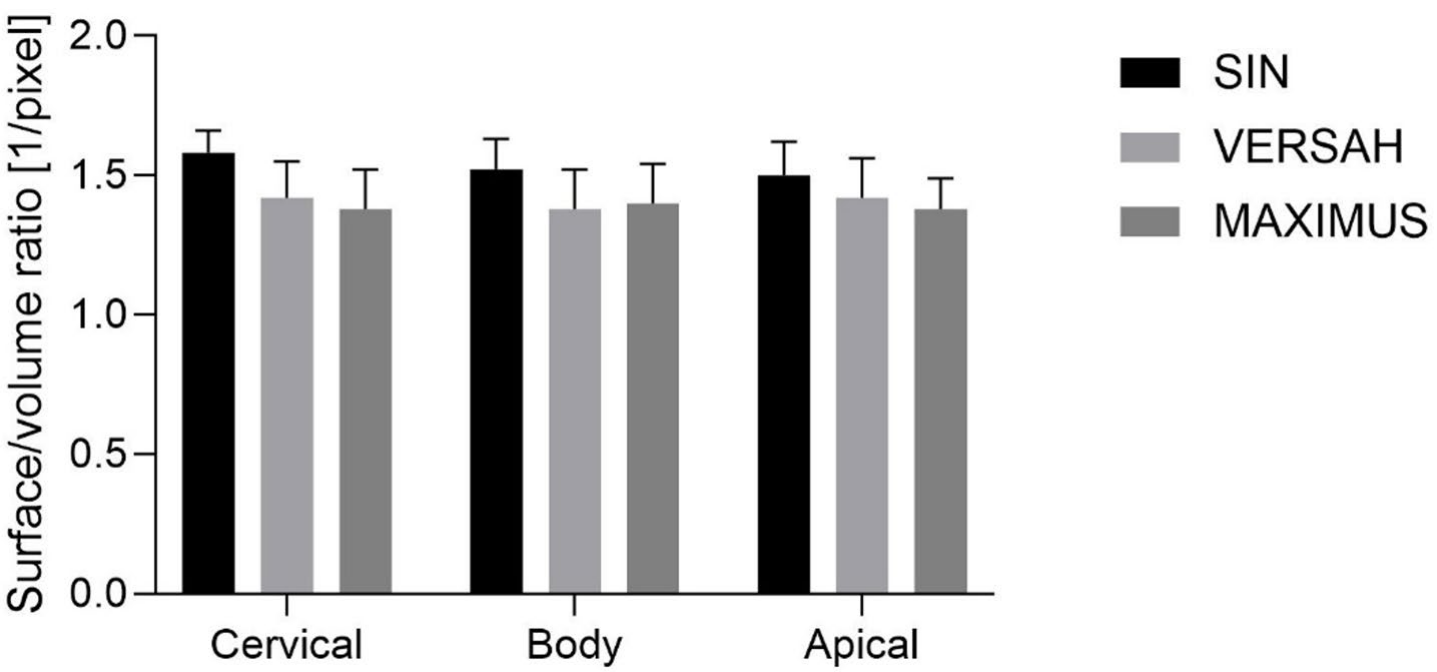
Bone microarchitecture results.

The bone surface/volume ratio was calculated, revealing only minor differences among the investigated regions for each milling technique. Compared with VERSAH and MAXIMUS, SIN demonstrated a tendency toward a higher surface/volume ratio, particularly in the cervical region. However, no statistically significant differences were observed between the milling techniques, irrespective of whether the cervical, body, or apical regions were analyzed (Fig. 3; Supplementary Table 1).

### Insertion torque

**Fig. 4:**
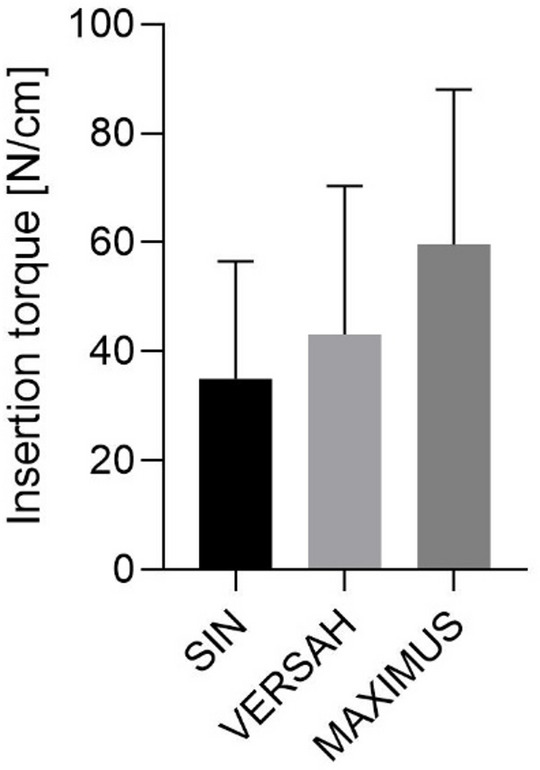
Insertion torque results.

Insertion torque was highest for the MAXIMUS milling technique, followed by VERSAH and SIN. Nevertheless, no statistically significant differences in insertion torque were detected among the three groups (Fig. 4; Supplementary Table 2).

## Discussion

In this in vitro study on low-density bone, our findings demonstrated that the three osteotomy techniques (SIN, VERSAH/osseodensification, and MAXIMUS/expanders) produced very similar bone microarchitecture, as indicated by the bone surface/volume ratio, and no statistically significant differences were observed in insertion torque (SIN: 35 ± 21.5 N cm; VERSAH: 43.2 ± 27.1 N cm; MAXIMUS: 59.6 ± 28.5 N cm; *p* > 0.05). These results suggest that, in low-density bone, the ability to modify the osteotomy site through densification may be limited by the biological properties of highly porous trabecular bone.

Previous studies have reported benefits of osseodensification in enhancing primary stability in low-density bone. For instance, Barberá-Millán et al. (2021) found that implants placed in low-density porcine tibia prepared with osseodensification exhibited significantly higher insertion torque and RFA values compared to conventional under-drilling (8.87 ± 6.17 N cm vs. 21.72 ± 17.14 N cm; ISQ 65.16 vs. 69.75)^17^. Similarly, a prospective clinical study by Hindi & Bede (2020) demonstrated that osseodensification led to a significant increase in peri-implant bone density (measured by CBCT) after implant placement, although a transient decrease in stability was observed during the first 6 weeks, followed by recovery at week 12^18^.

Conversely, other studies have highlighted limitations of osseodensification in very low-density bone. Although ovine models showed increased insertion torque with densification, not all histomorphometric parameters were significantly improved compared to conventional drilling^19,20^. Moreover, systematic reviews focusing on low-density regions, such as the posterior maxilla, indicate that osseodensification tends to increase ISQ values relative to conventional drilling, but the magnitude of this effect varies between studies, suggesting that outcomes depend on bone type, surgical protocol, and implant macrogeometry^21^.

Our finding of no statistically significant differences, despite numerically higher mean values for the densification systems (VERSAH and MAXIMUS), may reflect these intrinsic biological limitations of low-density bone. In highly porous bone, lateral and apical compaction achieved with osseodensification instruments may be limited or induce microdamage to trabeculae, reducing the expected mechanical advantage. Previous studies have warned that excessive torque can cause microfractures or local necrosis, compromising long-term primary stability^22^. Additionally, a recent in vivo study demonstrated that osseodensification can create wider osteotomies (“healing chambers”) without compromising primary stability in low-density bone, suggesting that space creation or subsequent remodeling may compensate for mechanical densification depending on the surgical strategy^21^.

These findings highlight that, in extremely low-density bone, the choice of osteotomy technique may have less impact than previously assumed, and local bone biology becomes the predominant factor determining primary stability. Clinically, this implies that simply using densification instruments may not be sufficient to achieve high insertion torque or optimal stability in these scenarios. Implant macrogeometry, conservative loading protocols, and adjunctive strategies—such as grafting, progressive compaction, or bone augmentation—may be more relevant for optimizing initial implant performance^23–27^.

Although the manufacturer recommends placing the implants 1.5–2.0 mm subcrestally, in the present study the implants were positioned at bone level. This decision was made to standardize the experimental conditions and to allow a more direct comparison of insertion torque values among the different osteotomy protocols. Subcrestal placement may increase the amount of bone–implant contact during insertion, potentially leading to higher insertion torque values due to additional friction from the crestal cortical bone. Therefore, placing the implants at bone level minimized the influence of crestal bone engagement on torque measurements, allowing the insertion torque to be primarily related to the preparation technique and implant design. This standardized approach improves the internal validity of the study, although it should be acknowledged that the results may differ from those obtained under clinical conditions following the manufacturer’s recommended subcrestal placement.

Future studies should explore combinations of osseodensification with other interventions to enhance local bone strength and assess effects not only on primary stability but also on secondary stability and bone remodeling over the medium and long term. Such investigations will clarify the extent to which osteotomy modification can compensate for intrinsic limitations of low-density bone and guide evidence-based clinical decision-making.

Overall, our results complement existing literature by demonstrating that, under highly unfavorable bone density conditions, the benefits of different osteotomy techniques may be less pronounced than suggested by optimistic studies. This does not imply that densification techniques are ineffective, but rather that their effect may approach a “threshold of utility” in extremely porous bone, where the biological architecture of the bone may become the limiting factor rather than the choice of drill.

## Conclusion

These results indicate that, under conditions of low bone density, the choice of milling technique may have a limited impact on altering bone microarchitecture or enhancing insertion torque. The similarity observed across conventional drilling, osseodensification, and bone expander approaches suggests that intrinsic properties of the trabecular bone may play a more dominant role in determining primary stability than the specific preparation method. This highlights the importance of considering bone quality alongside surgical technique when planning implant placement in low-density sites.

## Supplementary Information

Below is the link to the electronic supplementary material.

Supplementary Material 1

Supplementary Material 2

## Data Availability

The datasets used and/or analyzed during the current study available from the corresponding author on reasonable request.
