# Supplementary material for: In vitro evaluation of different implant systems and their influence on primary stability
Source: Sci Rep. 2026 Jan 9;16:1297. doi: 10.1038/s41598-026-35112-5 (PMC12791134; doi:10.1038/s41598-026-35112-5)
Supplement: Supplementary file 1 — Supplementary Material 1 [file 41598_2026_35112_MOESM1_ESM.docx]

Supplementary Table 1. Bone microarchitecture results of different milling techniques.

| Region | Bone surface/volume ratio (1/pixel; mean ± standard deviation) | | | Multiple comparisons (adjusted p values) | | |
| --- | --- | --- | --- | --- | --- | --- |
|  | SIN | VERSAH | MAXIMUS | SIN vs. VERSAH | SIN vs. MAXIMUS | VERSAH vs. MAXIMUS |
| Cervical | 1.58 ± 0.08 | 1.42 ± 0.13 | 1.38 ± 0.14 | 0.1282 | 0.0521 | 0.8588 |
| Body | 1.52 ± 0.11 | 1.38 ± 0.14 | 1.4 ± 0.14 | 0.2477 | 0.3475 | 0.9684 |
| Apical | 1.5 ± 0.12 | 1.42 ± 0.14 | 1.38 ± 0.11 | 0.5787 | 0.3120 | 0.8697 |
