# Supplementary material for: In vitro evaluation of different implant systems and their influence on primary stability
Source: Sci Rep. 2026 Jan 9;16:1297. doi: 10.1038/s41598-026-35112-5 (PMC12791134; doi:10.1038/s41598-026-35112-5)
Supplement: Supplementary file 2 — Supplementary Material 2 [file 41598_2026_35112_MOESM2_ESM.docx]

Supplementary Table 2. Insertion torque results.

| Milling technique | N/cm (mean ± standard deviation | Multiple comparisons (adjusted p values) |
| --- | --- | --- |
| SIN | 35 ± 21.5 | vs. VERSAH: 0.8722  vs. MAXIMUS: 0.3240 |
| VERSAH | 43.2 ± 27.1 | vs. MAXIMUS: 0.5896 |
| MAXIMUS | 59.6 ± 28.5 | - |
